# Perceiving the representative surface color of real-world materials

**DOI:** 10.1038/s41598-023-33563-8

**Published:** 2023-04-18

**Authors:** Yan Zhang, Isamu Motoyoshi

**Affiliations:** Department of Life Sciences, The University of Tokyo, Tokyo, Japan

**Keywords:** Human behaviour, Colour vision

## Abstract

Natural surfaces such as soil, grass, and skin usually involve far more complex and heterogenous structures than the perfectly uniform surfaces assumed in studies on color and material perception. Despite this, we can easily perceive the representative color of these surfaces. Here, we investigated the visual mechanisms underlying the perception of representative surface color using 120 natural images of diverse materials and their statistically synthesized images. Our matching experiments indicated that the perceived representative color revealed was not significantly different from the Portilla–Simoncelli-synthesized images or phase-randomized images except for one sample, even though the perceived shape and material properties were greatly impaired in the synthetic stimuli. The results also showed that the matched representative colors were predictable from the saturation-enhanced color of the brightest point in the image, excluding the high-intensity outliers. The results support the notion that humans judge the representative color and lightness of real-world surfaces depending on simple image measurements.

## Introduction

The world is full of objects with a variety of colors. Using information about color, we can recognize object categories, estimate the physical state of objects, communicate with other people using color names, and artificially create objects with a specific color^1,2^. All these behaviors rely on neural information processing in the visual system that estimates the color of objects.

The mechanisms by which the human eye and brain perceive the color of objects have been studied under the assumption that color perception is defined as a problem of estimating the physical reflectance properties of a surface. Early studies on "surface color" perception have investigated the perception of flat uniform color patches placed within a context such as a Mondrian background^3–6^ or regions within a simplified object such as sphere and cube (e.g.^7–9^). These studies demonstrated that humans could perceive constant color against large variations in color signals on the retinal image due to the intensity and wavelength distribution of illumination; i.e., color constancy^10–13^. However, these experimental data and theories can only deal with the color of matte, smooth, and uniform surfaces under simplified illuminations. Such surfaces are far from real-world objects, which have complex optical properties and shapes.

Recent studies on "material perception" approach the mechanism of color perception for more realistic objects under natural lighting^14,15^. Typical psychophysical experiments examine the perception of the apparent lightness and color of surfaces using 3D objects with various material properties generated by physically based computer graphics^16–19^ or photographs of objects made of a particular material^20–22^. Unlike flat patches, these objects produce complex images due to shading, specular reflections, and sub-surface scattering. Despite this, it has been shown that humans can easily perceive both lightness and color, as well as gloss and transparency, using various low- and high-order cues in the images^14^. It can be said that these studies allow us to get much closer to the mechanisms of color perception in the real world.

However, we rarely encounter smooth and coherent objects made of perfectly homogeneous materials, such as those used in the above. Although computer generated objects look very realistic because they precisely simulate the optics, they are ecologically irrelevant stimulus sets with a strong sampling bias. Real-world surfaces (e.g., rocks, skin, fabric, hair, and food) usually involve a more complicated structure including fibers, granules, scratches, and layers. In addition, they are often not composed of uniform materials. For example, many surfaces such as bark and stones are combinations of visibly different material parts. Furthermore, most surfaces involve dust, dirt, or cracks. Regardless, we can easily and steadily judge the overall color of a surface in daily life, unless the surface is composed of distinctively different colors. For example, we can say "This apple is a slightly dark red" about an apple even though the apple's skin actually involves lightness/color gradations, yellowish stripes, dark spots, and dust specks. In terms of ethology, this is the typical color perception that humans perform in most daily situations.

The perception of "representative color" has largely been ignored in conventional color studies because color perception has usually been defined as the estimation of physical reflectance (and therefore its mechanism has been investigated using artificial stimuli with uniform and simplified properties). However, we can find recent studies that addressed this issue explicitly or implicitly^23–27^. For example, Milojevic et al. (2018) showed that human observers could easily categorize photographs of autumn leaves into a single color category^27^. Giesel and Gegenfurtner (2010) examined the perceived color of real objects using various kinds of materials, such as wool balls and crumpled paper with similar colors, as well as objects made from the same material that differed only in color, in relation to the reflection properties of objects and the characteristics of image features^24^. These studies suggest that observers focus on the brightest (except highlights) and most saturated parts of the image to judge the representative color of an object^23–25,28,29^. Other studies conducted in the context of texture/ensemble perception also examined how the visual system can estimate the "average color" of spatial patterns composed of chromatic dots following a particular probability distribution^30–33^. Although the perception of the average color of such artificial stimuli may not be directly related to the representative color perception of natural surfaces, these findings would be useful when analyzing the perception of representative colors in terms of image features.

To investigate the role of image features in the perception of the representative color with a wide range of natural surfaces, the present study carried out a series of color-matching experiments to measure the perceived representative colors for 120 real-world surfaces made of diverse materials (e.g., grass, fabric, hair, metal, gravel, powder) and for their synthetic versions: Portilla-Simoncelli (PS)-synthesized images^34^ and phase-randomized images. The results showed that the matching data for both types of synthetic stimuli were not significantly different from that for the original surfaces except for one sample, even though the perceived 3D shape and material properties in the synthetic stimuli were greatly impaired. We found that the matching data for 360 stimuli were predictable by the lightness and slightly oversaturated color at the brightest point in the image, excluding high-intensity outliers. The results provide further evidence for the critical role of simple image features in the perception of representative surface color.

## Experiment 1: natural surface images

We measured the perceived representative colors of 120 natural surfaces with a variety of materials, and analyzed the perceptual data with respect to the average lightness and color of the images.

### Methods

#### Observers

Ten naïve paid volunteers including one of the authors took part in the experiment (seven males, 23 years old on average). All observers had normal or corrected-to-normal vision and normal color vision. All the experiments were conducted with the permission of the Ethical Review Committee for experiments on humans at the Graduate School of Arts and Sciences, The University of Tokyo. All observers provided written informed consent. The study followed the Declaration of Helsinki guidelines.

#### Apparatus

Visual stimuli were generated by a PC and displayed on an LCD monitor. Owing to the situation of COVID-19, stimuli were displayed on LCD/OEL monitors (three BENQ XL2730Z, two BENQ XL2430T, BENQ XL2735_B, SONY PVM-A250, SONY PVM 2541A, BENQ XL2731K, and BENQ XL2746S) set up in the observers' own homes. The viewing distance was adjusted so that the pixel resolution was 0.97 min/pixel. As a result, the size of the uniform background varied among monitors (from 59.7 (W) × 33.6 (H) to 53.1 (W) × 29.9 (H) deg), and the overall uniform background was much wider than the target image (4.6 × 4.6°). The background mean luminance was in the range 44–116 cd/m^2^. All monitors had gamma-corrected luminance, and a frame rate of 60 Hz. The CIE x–y coordinates (x,y) of each gun (r:g:b) of the monitors were on average (r: 0.65, 0.33), (g: 0.33, 0.61), and (b: 0.15, 0.05) with standard deviations of (r: 0.008, 0.006), (g: 0.015, 0.011), and (b: 0.011, 0.011), respectively. These variations in chromaticity across monitors were much smaller than those of the matching data across observers.

#### Color space

The absolute luminance and color of the stimuli varied between observers because different monitors were used for each observer. If this variation was large, it would make our analysis (see the Results section) based on matching data averaged across observers difficult. To check whether the variation was sufficiently small, we calculated the s.d. of color signals across the monitors (10×) for all possible pixels in all surface images used (256 × 256 pixels × 120 images). The calculation was performed in the CIE L a* b* space, which was employed in our matching experiment (see “Procedure” section). The La*b* values were computed from the luminance values of each gun that were actually measured for each monitor. The average s.d. were 0.15 for L, 0.26 for a*, and 0.97 for b*. The analysis indicated that the variability of a* and b* were far smaller than those across observers (3.0 for a*, and 3.7 for b*) whereas the variability of L was relatively large as compared to that of the matched L across observers (s.d. of 0.19), probably reflecting a large variation in the absolute luminance level across individual monitors. In addition to these relatively small variability of color signals across monitors, it is also important to note that the present study is focused on the relationship in the color between the reference patch and the test stimulus on the individual monitor. Accordingly, we assumed that the pixels on each observer's monitor had a common luminance and color in sRGB space, and treated all stimuli and matching values in La^*^b^*^ space, which is transformed from sRGB space, by approximately defining them as having lightness and color in a common sRGB space. This made it possible, albeit imperfectly, to examine the relationship between test images and matched colors in the same space across all observers.

#### Stimuli

**Figure 1:**
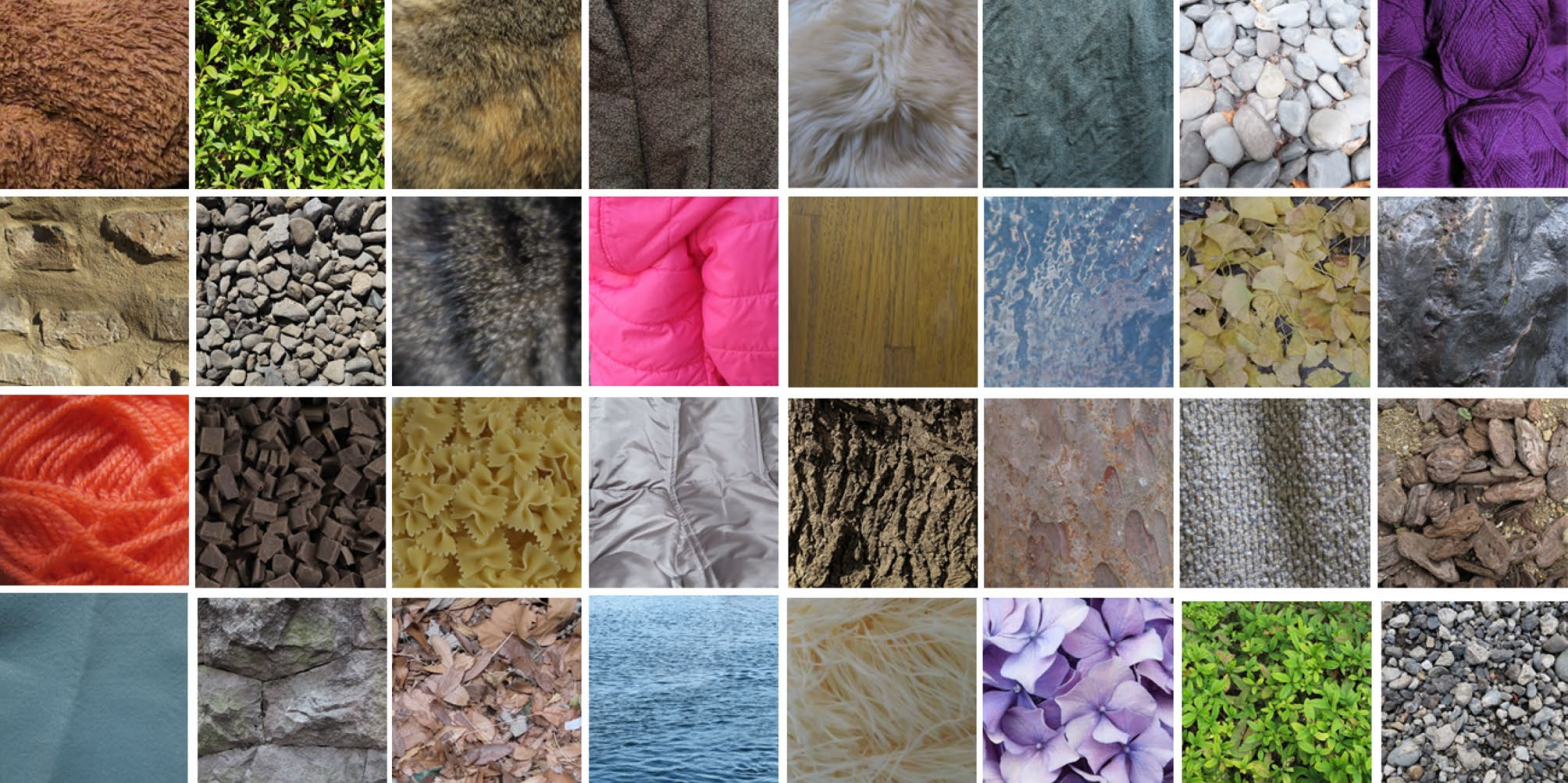
Examples of natural surface images used in Experiment 1.

Visual stimuli were photographed images of 120 natural surfaces (256 × 256 pixels, Fig. 1) including various materials such as stone, fabric, wood, grass, soil, mud, and metal. The images were taken from our own image database and other sites on the Internet. Images in which it is difficult to perceive a single representative color, such as surfaces with multiple regions of distinctly different colors or a set of objects with distinctly different color elements, were not used in the experiments.

**Figure 2:**
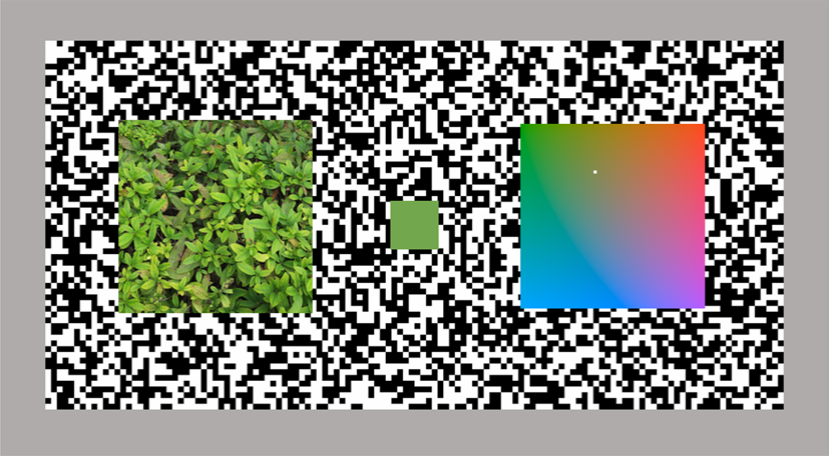
Schematic diagram of the stimulus display in Experiment 1.

As shown in Fig. 2, the visual display consisted of the test surface image on the left (4.6 × 4.6°), the uniform reference patch in the center (1.2 × 1.2°), and a color palette to assist the observer's color matching (4.6 × 4.6°). All of these were located on a black-and-white random dot background (30.2 × 15.1°), which enabled the matching to be effectively performed under constant adaptation or anchoring level across various test images. In the palette, a white dot was drawn to indicate the current a^*^–b^*^ coordinates of the reference patch that the observer was adjusting. The observers could make use of this dot as a guide to know which way to change the color of the reference patch, but they were strongly instructed not to refer the dot position to make a final decision.

#### Procedure

For each surface, the apparent representative color was measured by an asymmetric color matching task based on the adjustment method. Observers were asked to adjust both the lightness and color of the central reference patch so that it is perceptually best matched to the "representative color" of the entire natural surface. They were instructed to match the color that they usually answer in daily life when asked "what color is it?" but not to respond to the physical reflectance of a particular local part of the surface, nor to make use of any special knowledge about the physical material of the surface; they were asked, for example, not to imagine the color of the diffuse component excluding the specular reflection of a surface covered with a blurry gloss (e.g., skin, leather, leaves), nor to imagine the color of the dye used to dye the cloth (e.g., fabrics). In each trial, the observer binocularly viewed the display with free gaze and adjusted the lightness and color of the reference patch in the La^*^b^*^ space by pressing buttons. Observers adjusted a^*^ and b^*^ by pressing two horizontally and vertically paired buttons, respectively. Pressing the right or left button respectively increased or decreased a^*^ by 1.0, and pressing the up or down button respectively increased or decreased b^*^ by 1.0. The current (a^*^, b^*^) values were reflected in the position of the white cursor dot on the palette immediately. When observers pressed the up or down button of another pair of vertically aligned buttons, L was increased or decreased by 1.0. The current L value was reflected as the L value of the entire palette. The observers pressed the decision button when they considered the color and lightness of the reference patch to be satisfactorily close to the representative color of the test surface. Then, the (L, a^*^, b^*^) values of the reference patch were recorded. The observers were encouraged to adjust the color along the continuous color appearance without relying on the categorical color name. There was no time limit for matching, and observers spent an average of ~ 47 s with each stimulus. All observers practiced the matching task in advance in short sessions consisting of four or five trials.

It is more important to collect response data from as many diverse stimuli as possible to obtain ecologically valid findings than to collect accurate data from each observer by repeated measurements for a small number of stimuli. For this reason, the number of stimuli was prioritized in the present study over the accuracy of an individual observer's data. Therefore, each observer matched each test image once, but it took more than 1–1.5 h to complete all stimuli (a total of 3.0–4.5 h for the 360 stimuli in Experiments 1 and 2). For each test image, we obtained the mean and s.e.m. of the L, a^*^, and b^*^ values across ten observers.

### Results

**Figure 3:**
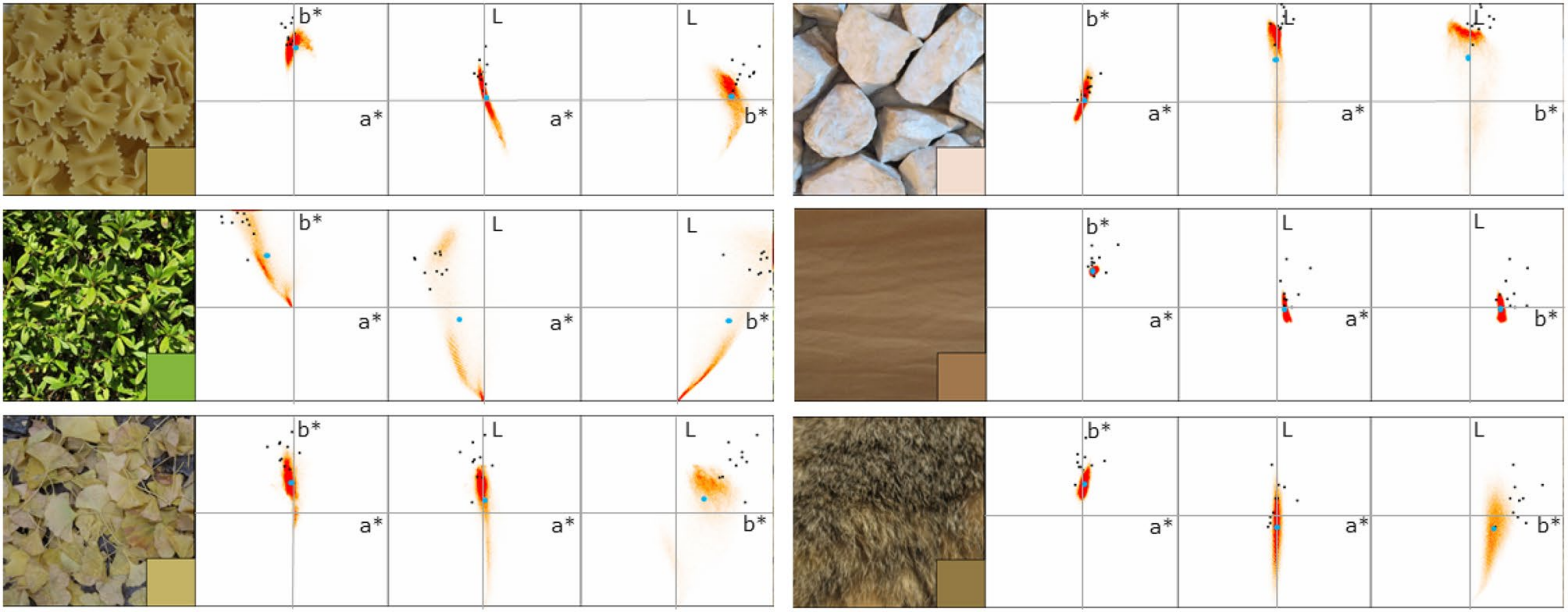
Examples of representative colors matched for natural surfaces. The test image is on the left of each panel. The lower right inset patch shows the matched lightness and color averaged across observers. The three scatter plots on the right represent the joint histogram of the test stimulus pixels plotted on the a^*^ vs. b^*^, L vs. a^*^, and L vs. b^*^ planes (red clouds), the between-observer average of the matched representative color (black dots), and the pixel mean of the test image (blue dots), respectively. Error bars represent the s.e.m. among 10 observers.

Figure 3 shows an example of the results obtained for six natural surfaces. On the left of each panel is the test image, and the inset on the lower right patch of each image is the matched representative color averaged across observers. The red clouds in the three scatter plots on the right show the joint histograms of pixels in the test images plotted in the a^*^–b^*^, L–a^*^, and L–b^*^ planes, respectively. The black dots indicate the matched color by individual observers. The blue dot represents the pixel-mean of the test image, which is merely introduced as a reference to visualize the trend of the matching data (we did not use the mode since we found it difficult to see the single global peak in the histogram for many images).

**Figure 4:**
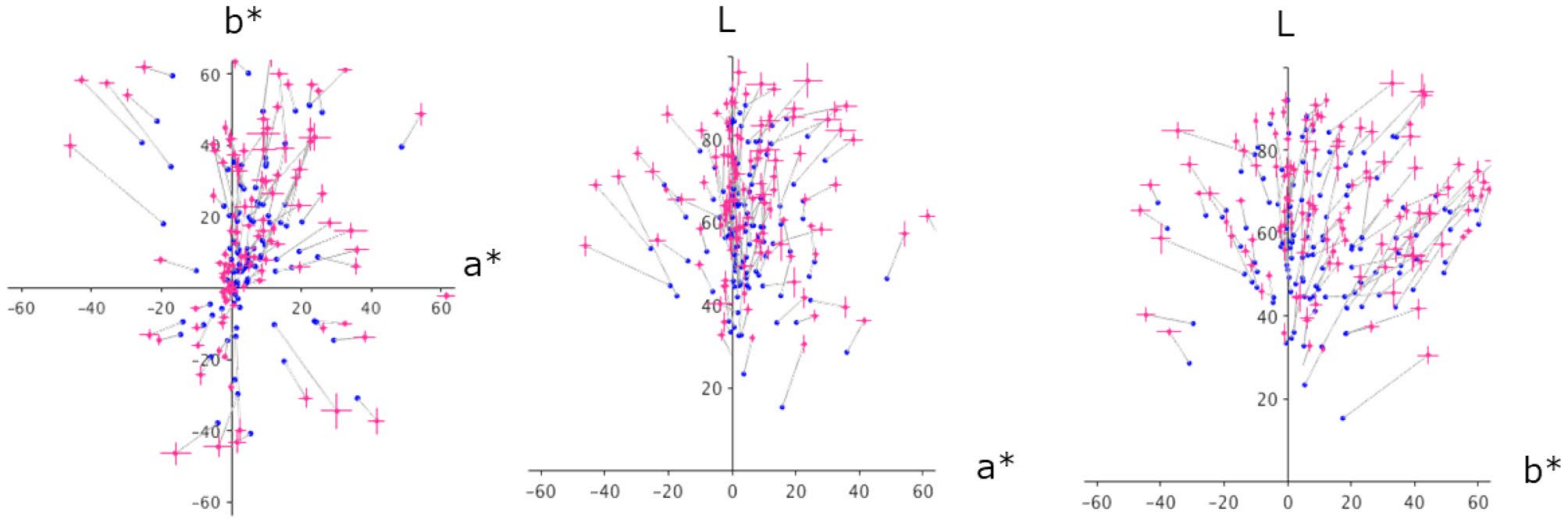
Distribution of matched representative colors in La^*^b^*^ color space. The red circles represent the matched representative colors averaged across 10 observers, and the blue circles connected to it represent the pixel mean of the test image. Each panel shows the results in the a^*^–b^*^, a^*^–L, and b^*^–L planes. Error bars represent the ± 1 s.e.m. across observers.

It is found that the mean matched colors (black dots) tend to deviate from the pixel mean of lightness and chromaticity of the image (blue dots). In Fig. 4, we plot the pixel mean of the image (blue circles) and the matched representative color for all test images (red circles), connected by lines. Each panel represents the result on the a^*^–b^*^, a^*^–L, and b^*^–L planes, respectively. For almost all test images, it is clear that the matched representative color deviates from the pixel mean. The matched lightness (L_match_) is higher than the mean lightness (L_mean_), and the matched colors in the a^*^–b^*^ coordinates are farther from the origin than the image mean, i.e., they are more saturated.

**Figure 5:**
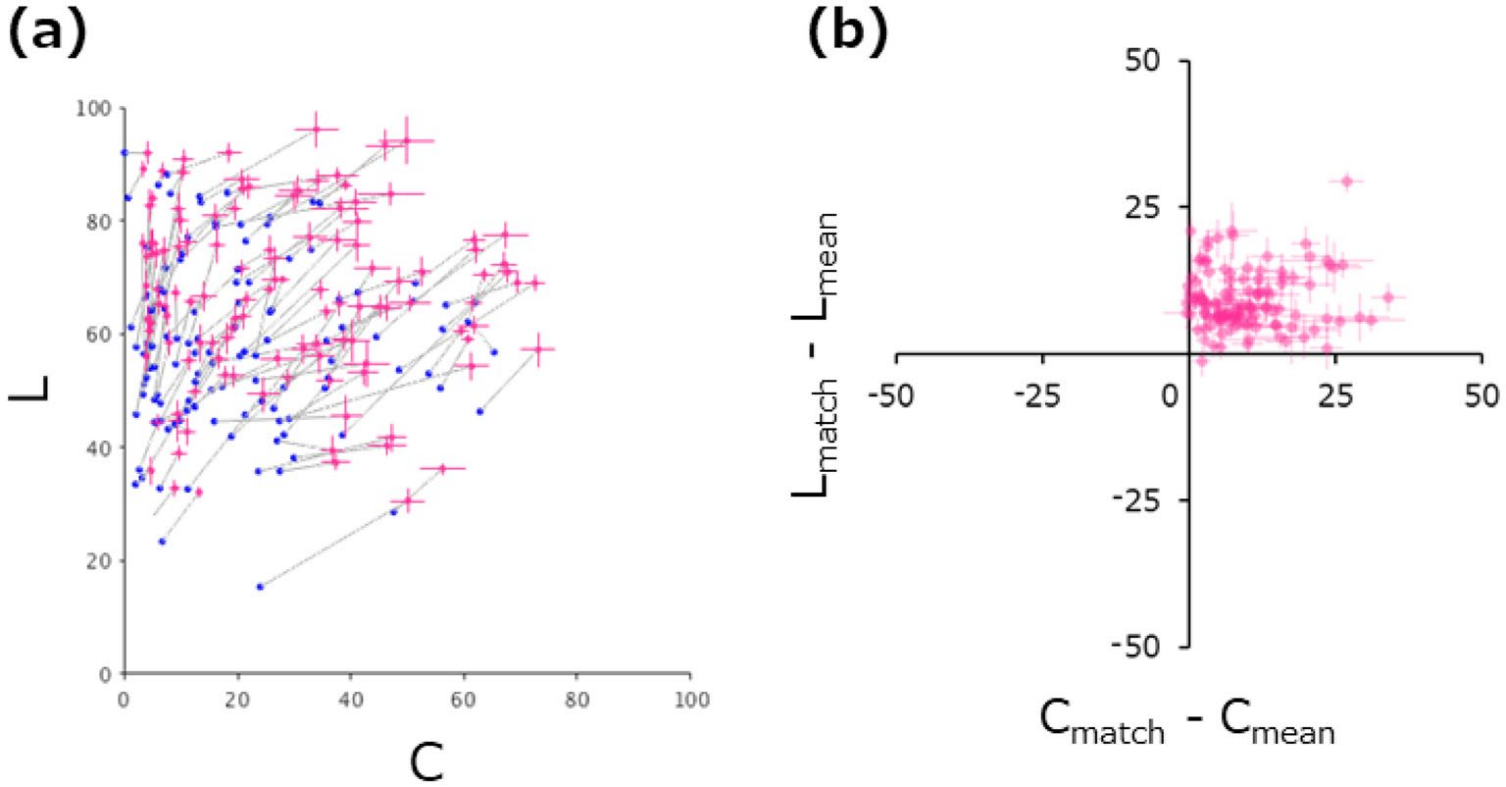
(**a**) Distribution of matched representative colors in the L-chroma plane. The red circles represent the matched representative color, and the blue circle connected to it by a line represents the pixel mean of the test image. (**b**) Difference between the matched representative color and the pixel mean of the image. Positive values indicate that the matched color had a higher lightness (Y-axis) and chroma (X-axis) than the image mean. Error bars represent the ± 1 s.e.m. across observers.

Figure 5a shows the data replotted on the plane of L and chroma (i.e., saturation) given by C = sqrt (a^*2^ + b^*2^). Figure 5b shows the difference between the matched representative color and the image mean. These plots demonstrate that for almost all images, the matched lightness (L_match_) and chroma (C_match_) are higher than the image mean (L_mean_ and C_mean_). We confirmed that, on average across images, L_match_ is significantly higher than L_mean_ (t(119) = 20.02, p < 0.0001) and C_match_ is significantly higher than C_mean_ (t(119) = 13.70, p < 0.0001).

These data indicate a tendency for observers to estimate lightness and saturation as higher than the image mean when matching representative colors for a variety of natural surfaces. Additional analysis revealed that for as many as 36% of the stimuli, the saturation of the matched representative color was higher than the maximum saturation of all pixels. This overestimation was not observed for lightness. Similar tendencies have been reported in previous studies. For example, it has been suggested that human observers perceive lightness by ignoring dark shadows and highlights in 3D objects^17,35^, and by ignoring brighter or darker tails in the skewed luminance histogram of natural surfaces^20,21^. Studies of ensemble color perception have reported that human observers perceive the "average color" of a chromatic random-dot pattern with a very enhanced saturation^31,36^, which is often beyond the maximum in the stimulus^30,37^. These agreements seem to suggest that the trend we observed in the current data is consistent with a general rule in the perception of representative (or average) colors for various classes of visual stimuli. Thus, this leads us to hypothesize that the perceived representative color of a natural surface is correlated with simple measurements of the image, as suggested by previous studies^23–25,28^.

## Experiment 2: synthetic texture images

To examine the above hypothesis directly, we next examined the perceived representative color for synthetic images such as Portilla-Simoncelli (PS)-synthesized images and phase-randomized images, which have image statistics equal or similar to those of the original images but with significant loss of 3D structure and material perception. If the perception of representative color solely depends on image features, then similar matching data would be obtained. On the other hand, if higher-order information beyond low-level image features is also important, then different matching data would be obtained.

### Methods

**Figure 6:**
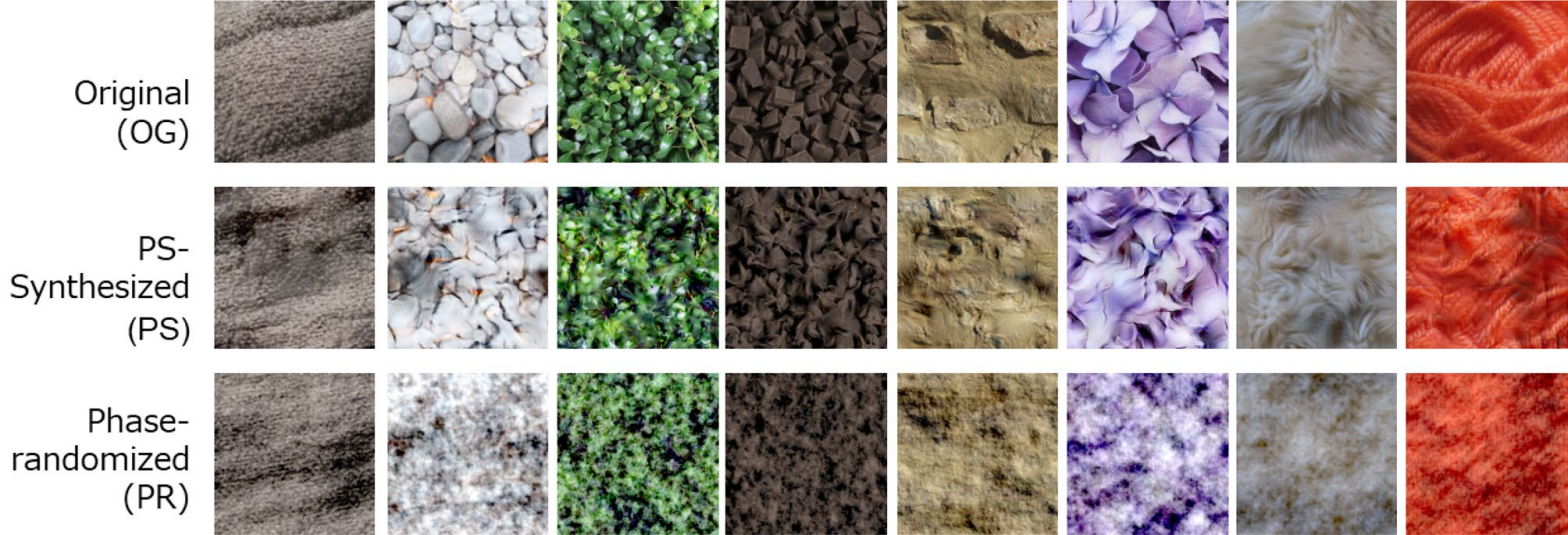
Examples of synthetic images generated from the originals. The top row shows the original image (OG). The middle row shows an image synthesized based on the Portilla–Simoncelli model (PS). The bottom row shows phase-randomized images (PR).

PS-synthesized images and phase-randomized images were created from the natural surface images used in Experiment 1. Figure 6 shows examples of the two types of synthetic images: the PS-synthesized image has nearly identical low- and high-level image statistics considered in the Portilla-Simoncelli's texture model with the original image^34^. Phase-randomized images are images that preserve only the global spatial-frequency spectrum of the original image. These synthetic images maintain a textural impression similar to the original images to some extent, but the perception of 3D shape and material properties are substantially impaired.

For all image stimulus, all observers participating in Experiment 1 were asked to match the representative color of the test image. All other conditions were the same as those in Experiment 1.

#### Ratings of material properties

In addition, to see if the perception of shape and surface properties is impaired in the synthetic images, we independently carried out a simple rating experiment. In each trial, the test image was presented in the center of the random-dot background, as in Experiment 1. Observers were asked to rate each of the three surface properties, glossiness, variation of depth, and softness, on a 5-point scale individually in separate blocks. Glossiness was defined as almost completely matte (0) to clearly shiny (4). Variation of depth was defined as almost completely flat (0) to very large variation (4) in the 3D depth of objects contained in the image, regardless of its physical origin such as bumpiness (e.g., crumpled paper) or overlaps between multiple surfaces (e.g., overlapping leaves in a tree). Softness refers to the ability of object(s) to be deformed, which includes elasticity, viscosity, and fragility, from being solid and fixed (0) to being very easily deformed (4) when touched. Each observer made one rating for each stimulus. The experimental blocks were independent for the original image, the PS-synthesized image, and the phase-randomized images. Nine of the observers who participated in Experiment 1 took part in the rating experiment.

### Results

**Figure 7:**
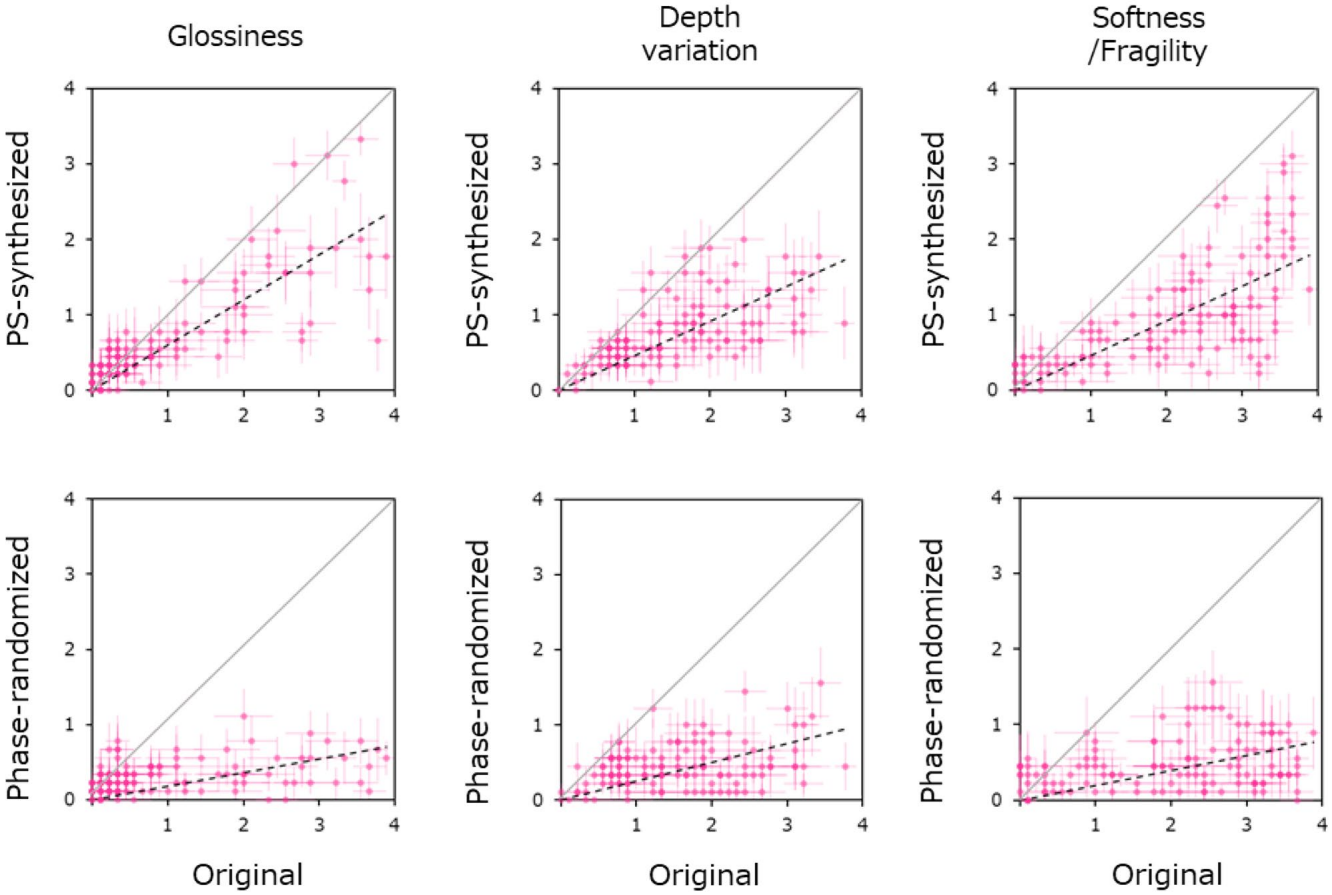
Surface properties ratings for the original and the synthetic images. Each panel shows the relationship between the ratings obtained for the original and the synthetic image. The left, middle, and right columns show the results for glossiness, depth variation, and softness, respectively. The upper panel shows the relationship between the original image and the PS-synthesized image, and the lower panel shows the relationship between the original image and the phase-randomized image. Error bars indicate the ± 1 s.e.m. across observers.

Figure 7 shows the relationship between the rating obtained for the original image (X-axis) and the rating obtained for the synthetic image (Y-axis). Each column shows the results for different surface properties. For all attributes, the ratings obtained for the PS-synthesized image (top row) are significantly lower than those obtained for the original image, and the ratings for the phase-randomized image are often reduced to near zero. The t-test statistics showed that ratings for the PS-synthesized images were significantly lower than the original image for all three attributes (glossiness: t(119) = 5.15, p < 0.0001; variation of depth: t(119) = 12.17, p < 0.0001; softness: t(119) = 13.20, p < 0.0001), and the same was true for the phase-randomized images (glossiness: t(119) = 7.46, p < 0.0001; variation of depth: t(119) = 14.59, p < 0.0001; softness: t(119) = 14.51, p < 0.0001).

These results indicate that the perception of surface properties is substantially impaired in the synthetic images, especially in the phase-randomized images, which is consistent with findings demonstrating the insufficiency of image statistics in material perception (e.g.^20,38,39^). Thus, if the perception of representative color depends on information beyond image statistics, it is strongly expected that the color matching for synthetic images would significantly differ from that for original images.

**Figure 8:**
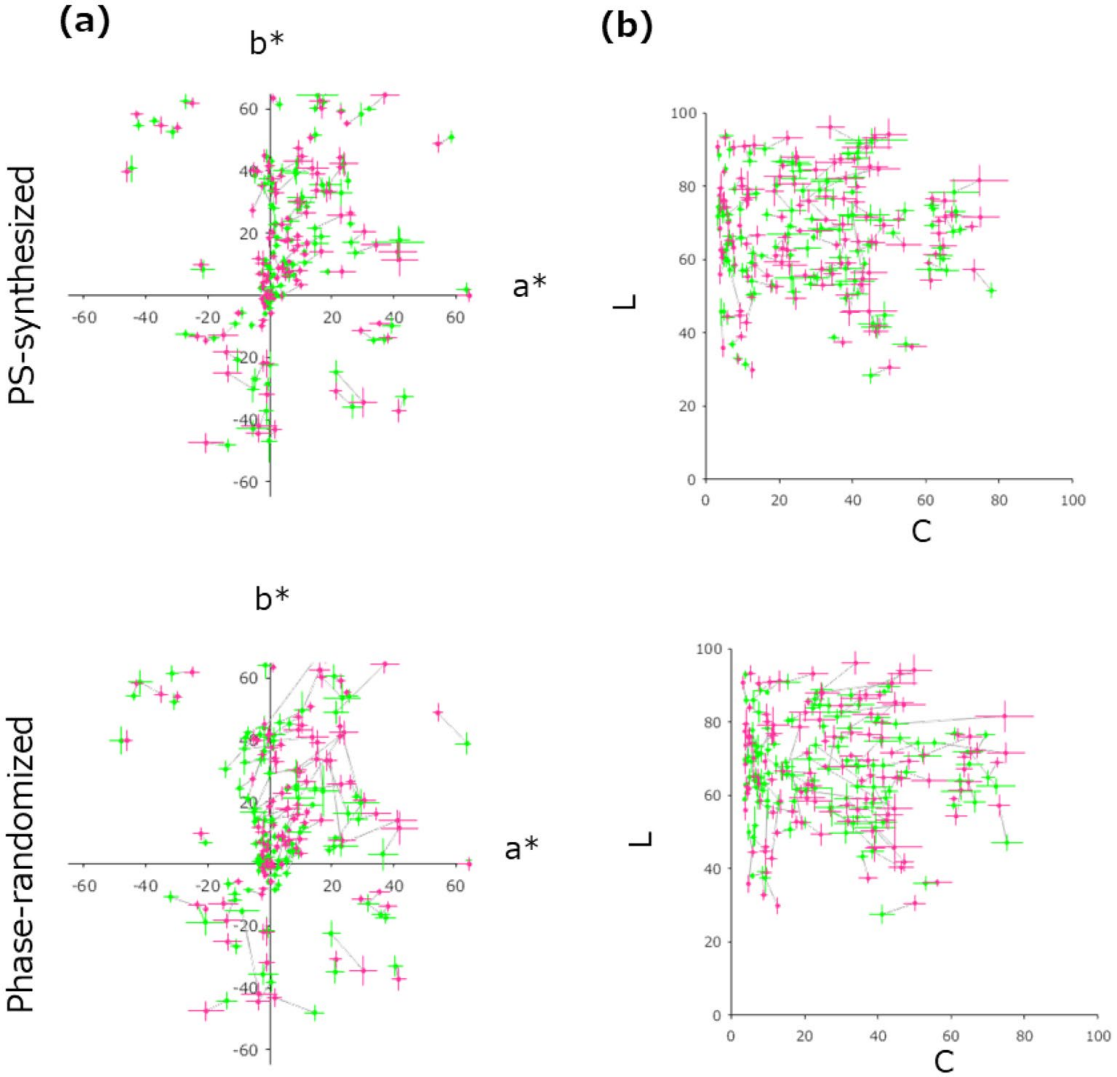
Matched representative colors for PS-synthesized images (upper panels) and phase-randomized images (lower panels). (**a,b**) Matched representative colors for the original and synthetic images. The green circle indicates the mean of the matched representative color of the synthetic images for 10 observers, and the red circle connected to it by a line indicates the mean of matched representative color for the originals: (**a**) shows the result in the a^*^–b^*^ planes, and (**b**) shows the result in the L-chroma planes.

Figure 8a shows the color matching results obtained for the synthetic images. The green circles show the matched representative colors plotted on the a^*^–b^*^ plane for the PS-synthesized image (top) and the phase-randomized image (bottom). The red circle connected by gray line indicates the representative color matched for the original image. Figure 8b shows a replot of the data in the L-chroma plane. Considering the errors across observers, these plots show that the matched representative colors for both synthetic images, especially for the PS-synthesized image, are not much different from those for the original images. In fact, the Pearson’s correlation coefficients were [L, a*, b*] = [0.97, 0.98, 0.99] (t(118) = 45.31: p < 10^–76^, t(118) = 60.61: p < 10^–91^, t(118) = 90.96: p < 10^–111^) for the PS-synthesized images, and [L, a*, b*] = [0.95, 0.95, 0.97] (t(118) = 31.56: p < 10^–59^, t(118) = 32.60: p < 10^–61^, t(118) = 40.71: p < 10^–71^) for the phase-randomized images, respectively.

More strictly, for each surface, we statistically tested the difference in the matched representative color between the synthetic image and original image, using the T^2^ test based on the Mahalanobis distance of data in the La^*^b^*^ space. As a result, we found no images whose matched color was significantly (p < 0.01) different from the original for the PS-synthesized images, and four images for the phase-randomized images. Applying multiple comparison correction [Benjamini–Hochberg’s FDR (0.05)], we found only one image showing a significant difference among 240 pairs. Thus, we failed to find clear evidence that observers judged the representative color of a natural surface in different ways than they do for synthetic textures with similar image/color statistics. Instead, the results support the idea that the perception of representative colors depends on low-level image measurements, in contrast to the perception of the other material properties. It should be noted that these results are obtained for 120 real-world surfaces with much larger variations and higher ecological validity than those used in most of the previous studies^5,7,8,13,19,30,31,36,37^.

## Experiment 3: image-based model

The results of two experiments show that perceived representative colors of natural surfaces were generally higher in lightness and saturation than the mean of the image, and were not different from those for the synthetic images, except for one phase-randomized image. This supports the idea that the human visual system utilizes simple image features to estimate the representative color of a natural surface. What image features are diagnostic of the representative color?

As noted earlier, previous studies, although with a limited class of objects, support the notion that the perceived representative color corresponds to the brightest and most saturated color in the image area, excluding dark shadows and bright specular highlights^25,28–30,35,37,40^. Given the present finding that the perceived representative color did not differ in synthetic images (except 1 out of 240 images) where shadows and glossy highlights were not properly perceived, it is more reasonable to assume that the visual system merely excludes the outliers with extremely high intensity, irrespective of whether they are specular highlights or not. In addition, in accord with the fact that the saturation of the matched representative color was often higher than the maximum saturation in the image, it is likely that the visual system overestimates saturation in the judgement of the representative color; similar oversaturation has been reported in previous studies^24,30,37,40^. With reference to these findings, we have developed a simple model in which the perceived representative color is determined solely by (1) the (L, a*, b*) value of a pixel with the highest L within the image excluding the higher outliers in the image intensity (L) distribution and (2) the saturation (chroma) enhancement.

Specifically, the model extracts the region in which the contrast value C(x,y) = L(x,y)/L_mean_ − 1 of the image L(x,y) is not higher than the threshold C_threshold_, and picks the La*b* value of a pixel with the highest L within the region, then multiplies a* and b* by M. The resulting (L, a^*^, b^*^) value is then assumed to be matched as the representative color of that surface image. We conducted a numerical simulation in which the two parameters (C_threshold_, M) were optimized to minimize the distance between the La^*^b^*^ value predicted by the model and the average La^*^b^*^ value by each human observer. We found that the optimized parameters were (C_threshold_, M) = (0.20, 1.26).

**Figure 9:**
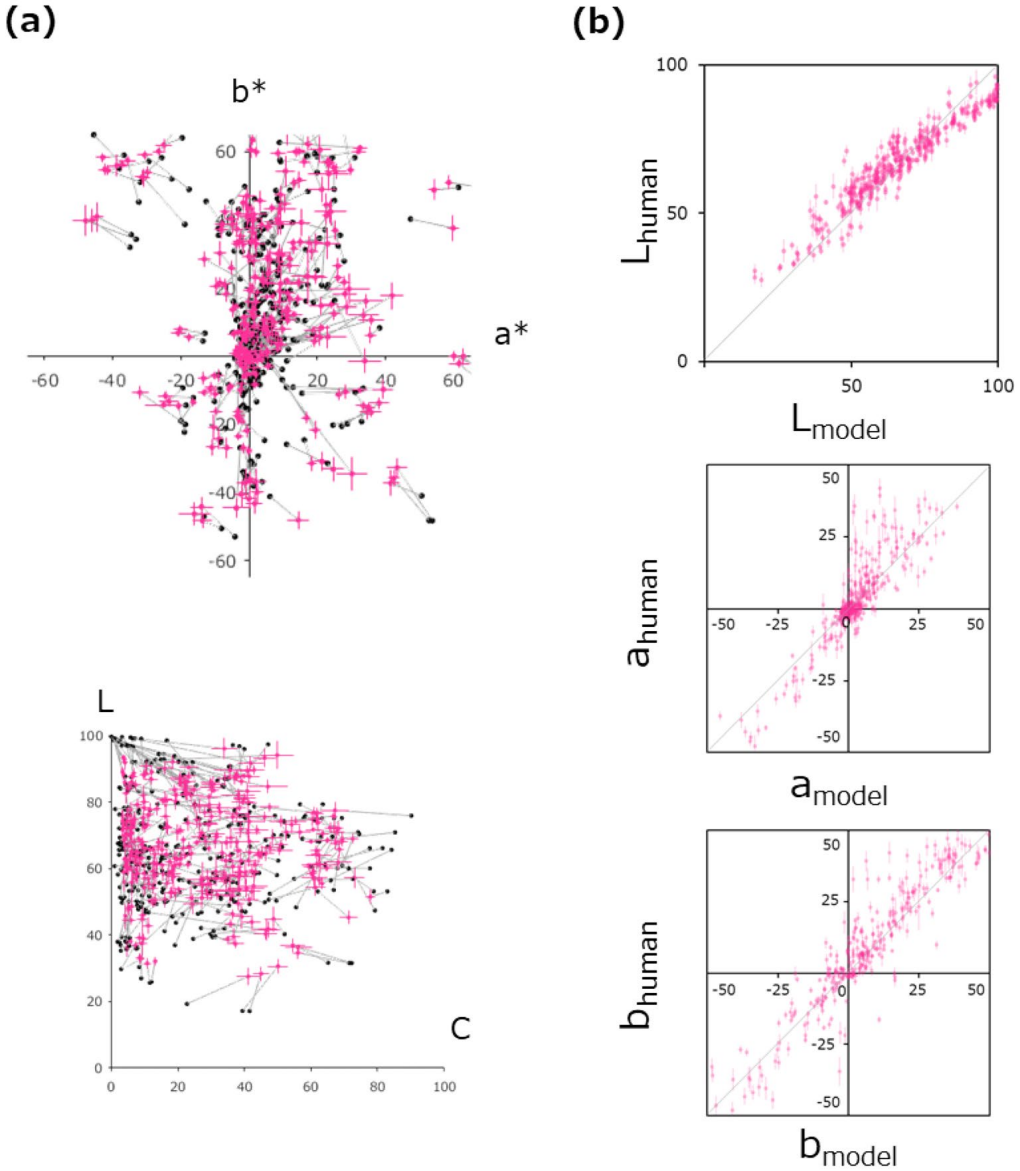
(**a**) Matching data predicted by the model and observed data. Results for all 360 natural and synthetic images. A red circle represents the average of the matched representative color, and the black dot connected to it by a line represents the color predicted by the model. The upper and lower panels show the results in the a^*^–b^*^ and L-chroma planes, respectively. (**b**) The relationship between the human data and model data. The horizontal axis shows the model predictions, and the vertical axis shows the human data. The top, middle, and bottom panels show the results for the L, a^*^, and b^*^ axes, respectively. Error bars represent the ± 1 s.e.m. across observers.

Figure 9a plots the model predictions and the human matching data in the a*-b* plane (top panel) and the L-chroma plane (bottom panel) for all 360 images used. Figure 9b plots the model (X-axis) and human (Y-axis) matching data in the L, a*, and b* dimensions individually. The Pearson’s correlation coefficients between the observed and predicted data were [L, a*, b*] = [0.95, 0.90, 0.95] (t(358) = 59.88: p < 10^–188^, t(358) = 38.35: p < 10^–129^, t(358) = 55.74: p < 10^–178^). A T^2^ test based on the Mahalanobis distance of data in the La^*^b^*^ space for the individual images showed that for 19 out of 360 images, the predicted data were significantly different (p < 0.01) from the human data (no images were significant if multiple comparisons were FDR-corrected). These results suggest that the representative color of stimuli, including natural and synthetic images matched by human observers, can be predicted by very simple pixel statistics solely. Although the prediction may appear to be still inaccurate, it is easily expected that models incorporating the other shallow and/or deep image features^34,41,42^ would make more accurate predictions without considering high-level conceptual features such as shape, occlusion, and highlights.

## Discussion

The present study investigated the characteristics and mechanisms of representative color perception for 120 kinds of real-world surfaces, most of which involved highly complex structures and non-uniform material properties. The results showed that observers matched representative colors with higher lightness and saturation than the mean of the image, which is partially consistent with previous findings^24,25,28,31,36,37^. More importantly, the present study revealed that the perceived representative colors for the original images were not different from those for the PS-synthesized and from those for phase-randomized images except for one. The results support the idea that the human visual system utilizes simple image features to estimate the representative color of a natural surface. It is important to note that our observers were not asked to accurately estimate the physical diffuse reflectance, but to simply indicate the "representative" color of objects. This may be the fundamental reason why observers referred to simple image measurements to make the judgement.

The current model is descriptive, and it is unclear how the visual system utilizes image features (i.e., the brightest point in the image excluding highlights) to make behavioral judgments about representative colors. One possibility is that the visual system serially samples representative image areas while ignoring outliers, then picks up the local information to make a decision about the color of the entire image. Actually, previous studies have demonstrated the role of such serial samplings in lightness/color perception^25,43^. An important finding of the present study is that, even in this relatively time-consuming process, the visual system makes decisions using low-level image features without relying on information about the shape and material properties of external objects. Moreover, these image features are likely to be very simple, such as maximum luminance or saturation, which does not require spatial pattern analysis. This supports the idea that the perception of representative color depends on low-level 'non-spatial' image features, in contrast to the perception of other surface material properties such as gloss and bumps, which require texture statistics based on spatial frequency and orientation analysis and/or even higher-order information. On the other hand, the present results do not necessarily exclude the possibility that surface color perception involves high-level spatial processing, such as segmentation and contour formation. They may also be useful to ignore irrelevant parts (e.g., specular highlights, dust specks) in a more sophisticated fashion. Whatever the alternative model or hypothesis would be, however, it should be able to explain the perceptual data obtained for many ecologically valid stimuli (e.g., photos of 120 daily objects we used), but not only for artificially created stimuli that hardly or never exist in the real world (e.g., solid-shaped CG objects, flat paper).

In the present study, we did not use images for which it was too difficult to perceive one representative color. According to our casual observations, such images often consist of regions that obviously have more than one hue. Indeed, it has been shown that hues tend to be consistent within a single object in natural scenes^44,45^, and that the hue consistency can be used to segment objects in natural images^46,47^. The colorful mimicry found in the epidermis of certain animals and insects reduces the probability of perceiving themselves as a single object because of the different hue patterns^48,49^. According to these findings, it is natural for us to be unable to easily perceive the representative color of an image region that is not hue-consistent and therefore cannot be considered a single-object surface. However, this account, which is based on hue variation, cannot explain the difficulty of finding a single color on particular (but not rare) surfaces with distinct texture patterns that have a similar hue but clearly different saturation. There are also cases in which the perception of representative colors is not difficult by ignoring some different colors if that color has only a small area, for example, when the ground is visible in a small portion of a lawn. To summarize, it appears to be difficult to determine whether a surface has a single color on the basis of simple image features alone. Decisions may also be influenced by top-down mechanisms, such as spatial scaling of attention. At present, we can only rely on the subjective judgment of the observer as to whether a single representative color can be perceived with confidence.

## Data Availability

The datasets generated and analyzed during the current study are available from the corresponding author on reasonable request.
